# Books and Authors

**Published:** 1907-12

**Authors:** 


### BOOKS AND AUTHORS.

---

**Medical Jurisprudence, Forensic Medicine, and Toxicology.** By **R. A. Witthaus,** A.M., M.D., Professor of Chemistry, Physics and Toxicology in Cornell University; and Tracy C. Becker, A.B., LL.B., Professor of Criminal Law and Medical Jurisprudence in the University of Buffalo. Second edition. Vol. II. Octavo, pp. vii-1008. New York: William Wood & Co. 1907. (Muslin, $6.00; law sheep, $7.00, net prices).

This volume continues the subject of forensic medicine, in which the medical-legal consideration of wounds is taken up by George Woolsey; the medico-legal consideration of gunshot wounds is discussed by Roswell Park; the medico-legal relations of electricity are dealt with by W. N. Bullard; the medico-legal consideration of death from mechanical suffocation is set forth by D. S. Lamb; death from submersion is handled by Irving C. Rosse. The determination of survivorship from a legal standpoint is considered by Tracy C. Becker, and from a medical standpoint by John Parmenter.

Forensic medicine of a bio-thanological character begins with a consideration of abortion and infanticide, by J. Chalmers Cameron; then Mr. Becker, himself, discourses as to when medical examination of the living is permitted or required by courts of law. Pregnancy, labor, and the puerperal state is the subject of a section by J. Clifton Edgar; sexual incapacity is discussed by Irving C. Rosse; rape forms the title of a chapter written jointly by J. Clifton Edgar and J. C. Johnston; unnatural crimes are discoursed upon by Rosse, and railway injuries furnishes the closing theme which is written by W. B. Outten.

The comprehensiveness of this work together with the minutiæ of detail and voluminous references make it of the utmost value to every legal or medical student of jurisprudence. No lawyer can afford to do without it nor should any physician who goes into the courts fail to possess it.

---

**A Manual of Personal Hygiene.** By eminent specialists. Edited by **Walter L. Pyle,** M.D., Assistant Surgeon to the Wills Eye Hospital, Philadelphia. Third edition. 12mo of 451 pages, illustrated. Philadelphia and London: W. B. Saunders Company, 1907. (Cloth, $1.50 net.)

The favorable comments made by the JOURNAL on the first edition of this book were justified, we may assume, by the demand for subsequent editions within comparatively short intervals. A somewhat extended and careful examination of this edition confirms our previous judgment, and tempts us to say that it is a book that should be found in the library of every well-bred or intelligent family. It is not a book for physicians, though they may obtain many valuable hints from its pages, but in particular it is for the laity. It tells the average man or woman how to keep well by intelligent care of the body.

The first section of the book deals with the hygiene of the digestive apparatus, and is written by Professor Charles G. Stockton, of Buffalo. It is one of the more important subjects in the manual and is placed very properly at the beginning. No more competent person could have been selected for this topic than Dr. Stockton whose skill in the management of diseases of the alimentary tract is known far and wide.

The sections on physical exercise and domestic hygiene deserve careful study by people in general, and by those whose bodies and homes are out of condition in particular.

A number of illustrations serve to elucidate the text in a telling manner. We could wish that physicians would advise families whom they serve, to obtain and carefully read this splendid book from cover to cover. It would be one of the most effective ways, in our view, in which to exercise their most important function,—that of the prevention of disease.

---

**Surgical Diagnosis.** By **Daniel N. Eisendrath,** M.D., Adjunct Professor of Surgery in the Medical Department of the University of Illinois (College of Physicians and Surgeons). Octavo of 775 pages, with 482 original illustrations, 15 in colors. Philadelphia and London: W. B. Saunders Company, 1907. (Cloth, $6.50 net; half morocco, $8.00 net.)

A good reason exists for the creation of this book, which is more than can be said of every work that is presented to the medical profession for its approval. Surgical diagnosis is even more important than medical, and no good reason can be offered against discussing it in a separate treatise, as has been done by several authors in the field of medical diseases. Eisendrath is well fitted for the task he has undertaken, being an excellent anatomist first, a skilful surgeon next, and a clinician last, while to these qualifications may be added those of a teacher of thoroughness and an author of accomplishment.

The arrangement of topics is made according to the author's own plans, differing from the usual manner in that diseases and injuries are grouped as considered when examination is made for diagnosis. We can see great advantage in considering together the injuries and diseases of each region or cavity of the body; especially does it facilitate clinical study for diagnostic purposes. So, also, does it materially aid in the differentiation of maladies or injuries which present many symptoms in common.

The discreet surgical teacher nowadays recognises the value of illustrations and this author has displayed good judgment in this regard, not only in the number but also in the quality of the engravings he has employed. As a final remark we asseverate that this is a treatise which no surgeon can afford to do without, while every general practitioner will find it a useful aid in his daily work.

---

**The Medical Record Visiting List or Physicians' Diary** for 1908. New Revised Edition. New York: William Wood & Company, Medical Publishers, 1908.

Year after year, with the regularity of the cycles, this splendid physicians' dairy is issued and becomes available for the convenience of physicians everywhere throughout the country. Its contents are elaborate and embrace the following: Calendar, estimation of the probable duration of pregnancy, approximate equivalents of temperature, weight, capacity, measure, etc., maximum adult doses by the mouth, in apothecaries' and decimal measures, drops in a fluid drachm, solutions for subcutaneous injection, solutions in water for automisation and inhalation, miscellaneous facts, emergencies, surgical antisepsis, disinfection, dentition, table of signs, visiting list with special memoranda, consultation practice, obstetric engagements, record of obstetrical practice, record of vaccination, register of deaths, nurses' addresses, addresses of patients and others, cash account.

The prices of regular lists are for 60 patients a week, with or without dates, handsomely selected red or black, morocco binding, $1.50; 30 patients a week, with or without dates, same style, $1.25; also a few special 90 patients lists, $2.00.

Among the novelties are published also visiting lists fitted into genuine seal and calf skin wallets, making most elegant books. The lists proper are in two books of six months each, and are removable from the wallets. They are thus much less bulky for the pocket, and are also economical inasmuch as the leather covers may be made to do service for several years. It is difficult to imagine a more useful book than this especially in such compact form, and for so reasonable a price.

---

**The Practitioners' Visiting List** for 1908. An invaluable pocket-sized book containing memoranda and data important for every physician, and ruled blanks for recording every detail of practice. The Weekly, Monthly and 30-Patient Perpetual contain 32 pages of data and 160 pages of classified blanks. The 60-Patient Perpetual consists of 256 pages of blanks alone. Each in one wallet-shaped book, bound in flexible leather, with flap and pocket, pencil and rubber, and calendar for two years. Price by mail, postpaid, to any address, $1.25. Thumb-letter index, 25 cents extra. Descriptive circular showing the several styles sent on request. Lea Brothers & Co., Publishers, Philadelphia and New York.

The foregoing description of this beautiful visiting list is so complete as to leave little to add. We may say, however, that we have rarely seen so useful or handsome a book for the purpose intended.

It contains among other valuable information a scheme of dentition; table of weights and measures and comparative scales; instructions for examining the urine; diagnostic table of eruptive fevers; incompatibles, poisons and antidotes; directions for effecting artificial respiration; extensive table of doses; an alphabetical table of diseases and their remedies, and directions for ligation of arteries. The record portion contains ruled blanks of various kinds, adapted for noting all details of practice and professional business.

The book is printed on fine, tough paper suitable for either pen or pencil, and bound with the utmost strength in handsome grained leather, and is sold at the lowest price compatible with perfection in every detail. It is one of the time-savers that no physician in active practice can afford to do without.

---

**A Manual of the Diagnosis and Treatment of the Diseases of the Eye.** By **Edward Jackson,** M.D., Professor of Ophthalmology in the University of Colorado. Second edition. 12mo of 615 pages with 182 text-illustrations and 2 colored plates. Philadelphia and London: W. B. Saunders Company, 1907. (Cloth, $2.50 net.)

To those acquainted with Professor Jackson's skill as an ophthalmologist very little need be said concerning this manual, which became so well and favorably known through its first edition. We thought then it was one of the best books of its class published up to that time. Some years, however, have passed since that time, and necessarily changes in teaching and practice have occurred. The author has incorporated all such that seemed of value, while avoiding those which only offered temporary attraction and bid fair to disappear completely after a little brief existence.

The newer methods of diagnosis that have been incorporated in this edition makes a somewhat lengthy list, yet the size of the book remains nearly the same, some eliminations and condensations having taken place. The illustrations are excellent and the text is full of practical instruction. It furnishes the student with a superb medium for acquiring that kind of instruction so needful at the outset of his studies in this field, while the general practitioner will find it most admirably adapted to his needs as a guide and reference book.

---

**A Textbook of Materia Medica for Nurses.** Including Therapeutics and Toxicology. By **George P. Paul, M.D.,** Assistant Visiting Physician and Adjunct Radiographer to the Samaritan Hospital, Troy, N. Y. 12mo of 240 pages. Philadelphia and London: W. B. Saunders Company, 1907. (Cloth, $1.50 net.)

To prepare a suitable book on this subject for nurses is not an easy task. Such an author must have had an experience, if not in teaching nurses, at least in directing them in the clinic. There is much to put into such a book and something to leave out, to be sure, and he is a fortunate author who makes no mistake on either side of this arbitrary line. We are pleased to observe that Paul has used excellent judgment both in what he has included in this book and in what he has omitted.

The material is arranged in six parts, the first consisting of general considerations; the second dealing with general materia medica, therapeutics and toxicology; the third describing drugs of minor importance; the fourth presenting the newer medicinal agents; the fifth offering practical therapeutics; while the sixth is an addendum giving the strength of drug preparations, synonyms, and weights and measures.

Each of these subdivisions possesses an importance peculiar to itself, and all are welded into a homogenious whole with skill and effectiveness. The definitions are concise yet adequate. The entire book betrays a care in preparation that is especially commendable in these days of hurry and hustle, which oftentimes provoke inaccuracy not only of expression but sometimes also misstatement of facts. No nurse who studies this book carefully will ever regret it.

**Modern Surgery: General and Operative.** By **J. Chalmers DaCosta,** M.D., Professor of the Principles of Surgery and of Clinical Surgery in the Jefferson Medical College, Philadelphia. Fifth edition. Octavo, 1283 pages, with 872 illustrations, some in colors. Philadelphia and London: W. B. Saunders Company, 1907. Cloth, $5.50; half morocco, $7.00 net prices.)

Like most authors who write a popular work on surgery, DaCosta has kept this treatise abreast of the times through frequent revisions. This edition gives evidence that the author has gone over the book with care, changing much of the old text, correcting and expanding it here and there and adding considerable new material until now it presents one of the most complete and useful single volumes on surgery that has been issued in this country. The number of sections in which changes have been made is twenty; and new material has been added to the number of thirty topics; also, many new illustrations have been incorporated.

DaCosta writes in epigrams that are remarkable for clearness and forceful truths. Scarcely a word is wasted in the entire volume, the result being that vast information is presented in the 1,300 pages that comprise the book.

The practical surgeon is portrayed in every chapter of this work, hence very little time is taken up with theories. It is doubtful if a better treatise on surgery exists in English today, and it is quite certain that as a textbook for both student and practitioner it is not excelled.

---

**Transactions of the American Dermatological Association** at its thirtieth annual meeting held at Cleveland, May 31, June 1, 2, 1906. **Grover W. Wende,** M.D., Secretary.

The transactions of this association, one of the oldest of the national special medical societies, always possess great interest for the specialist, while much of the material may be utilised by the general practitioner of medicine. This volume is no exception to the rule in these respects, and there are some features of the book that we have not seen heretofore incorporated in medical society transactions. Dr. Grover W. Wende, the secretary, who also is editor of the transactions, has added a list of photographs exhibited by the members, together with statistical returns for the year 1905, and a list of publications by the members for the year ending June 1, 1906. On the third day of the meeting a clinical session was held at the Lakeside Hospital, Cleveland, during which numerous interesting cases were shown and discussed. Dr. Wende himself presented a paper at the Cleveland meeting, reporting a case of infectious dermatitis gangrenosa. This unusual case is described at length in its clinical aspects, and the postmortem findings, too, are given in detail. A photograph of great clearness accompanies the report. This excellent volume deserves to be bound in cloth,—paper covers are poor preservatives of such good literature.

---

**The Care of the Baby.** By **J. P. Crozer Griffith,** M.D., Clinical Professor of Diseases of Children in the Hospital of the University of Pennsylvania. Fourth edition. 12mo of 455 pages, illustrated. Philadelphia and London: W. B. Saunders Company, 1907. (Cloth, $1.50 net.)

It is little wonder that this book is in its fourth edition. All in all it is doubtful if a better on the baby has ever been written. It tells the whole story with a clearness of diction that is praiseworthy and, besides, it has an underlying vein of common sense in every chapter, paragraph and sentence. Every woman about to become a mother can learn much she needs to know by a careful study of the first chapter, and after the baby comes she assuredly has a fund of information before her, that will serve as a guide in the care of her offspring from infancy to youth.

The author has carefully avoided the discussion of doubtful problems, hence gives conservative advice on infant feeding It is often a difficult question to determine, but Griffith handles it with discretion. We commend the book most heartily and wish that every mother capable of appreciating its contents could be induced to obtain it and be guided by its teachings.

---

**Report of the Commissioner of Education** for the year ending June 30, 1905. Vols. 1 and 2. Washington: Government Printing Office.

This report, embracing two standard octavo volumes is much decreased in size from previous reports, owing to the limitation of the appropriation for its publication, the amount being more than one-third less than formerly. It shows a decrease in the number of medical students of the country of 1,114 from that of the previous year, the total 25,835, being smaller than the number in any one of the preceding four years.

As usual these volumes are replete with valuable information for educators, and show a vast labor in their preparation.

---

**A Practician's Hand-Book of Materia Medica and Therapeutics,** based upon established physiologic actions and the indications in small doses. By **Thomas S. Blair,** M.D 250 pages, net. Published by The Medical Council, Philadelphia, Pa. (Price, $2.00.)

The author says this is an "exhortation," and we are inclined to think that he is right about it. We were in doubt where to place it until we discovered the author's own classification. However, it contains much valuable information about drugs and doses not easily found elsewhere, presented in excellent form, and with considerable acumen. It is one of the curiosities of drug literature and as such no doubt will prove entertaining to many readers.

---

**Atlas and Epitome of Diseases of Children.** By **Dr. R. Hecker** and **Dr. J. Trumpp,** of Munich. Edited, with additions, by **Isaac A. Abt,** M.D., Assistant Professor of the Diseases of Children in Rush Medical College (University of Chicago). With 48 colored plates, 147 black and white illustrations, and 453 pages of text. Philadelphia and London: W. B. Saunders Company, 1907. Cloth, $5.00 net.)

If proof were needed of the value of these Atlases the searcher would only find it necessary to examine this volume with care to settle the question. Rarely have we seen such beautiful plates, while many of the illustrations in the text are superb and all are excellent.

The junior practitioner can learn nearly as much from a careful study of this work as he can in the clinic, and we advise all such straightway to put themselves in possession of it. Skill in the treatment of diseases of children is not easily acquired, but this book will aid most satisfactorily in obtaining the requisite knowledge upon which success in this field can be predicated.

---

**The American Pocket Medical Dictionary.** Edited by **W. A. Newman Dorland,** M.D., editor "The American Illustrated Medical Dictionary." Fifth revised edition. 32mo, 574 pages. Philadelphia and London: W. B. Saunders Company. (Flexible morocco, gold edges, $1.00 net; thumb indexed, $1.25 net.)

A good small dictionary like this one is a convenience for a physician's office table one, we may be pardoned for saying, which he should always keep at hand. It is a time-saver, often filling a place for reference that answers quite as well as to take the trouble to consult a larger book. Dorland has constructed this edition on lines it would be difficult to improve upon. The words to be defined are in heavy-faced type while the definitions themselves are printed in sufficiently plain type to make it easy for even old eyes to read. The vocabulary is of wide range, adapted to the ordinary necessities of every-day use. The wonder is that so much of practical utility in the way of a word book has been put into such compact form. The book, too, in its make-up is a work of art.

---

**Twenty-sixth Annual Report of the State Department of Health of New York** for the year ending December 31, 1905. Albany: Brandow Printing Co.

These annual reports contain material of great value to sanitarians and public health officers. This one, especially, is fraught with interest containing as it does the address delivered at the fifth sanitary conference of the sanitary officers of the state. The discussion relating to water supplies in various parts of the state is always important, and so, too, is the account of what is doing in regard to sewers and sewage. If more study was given to these reports it would be better for the people.

